# Effectivity of Awareness Months in Increasing Internet Search Activity for Top Malignancies Among Women

**DOI:** 10.2196/publichealth.7714

**Published:** 2017-08-21

**Authors:** Dhruvika Mukhija, Anand Venkatraman, Sajan Jiv Singh Nagpal

**Affiliations:** ^1^ Cleveland Clinic Internal Medicine Cleveland, OH United States; ^2^ University of Alabama Neurology Birmingham, AL United States; ^3^ Mayo Clinic Rochester, MN United States

**Keywords:** colorectal cancer, lung cancer, breast cancer, cancer awareness month, infoveillance

## Letter

In a recent article, Ling et al. hypothesized that following the launch of a campaign for a medical condition, information seeking behavior pertaining to the condition would increase as well [1]. They used data from Google Trends (Google Inc., CA) on 4 different diseases (including Colon Cancer) to conclude that the use of infoveillance (type of public health surveillance based on online content analysis),shows promise as an alternative and inexpensive solution for disease surveillance and health care campaign evaluation. While a number of health campaigns are rolled out by the government and professional societies, there are limited means and tools to evaluate the effectivity of these campaigns. Using infoveillance, the ‘digital impact’ of various health- and disease-related campaigns can be assessed.

Cancer awareness has massively benefited from rapid growth of internet and mass media and the evolution of social marketing strategies around the promotion of healthcare [2,3]. This has resulted in the development of cancer oriented societies, websites, public campaigns and specifically earmarked Cancer Awareness Months (CAMs) directed at changing public attitudes towards prevention, screening, treatment and informed decision making. However, despite the significant impact of cancer awareness on screening of preventable cancers [3], the impact of CAMs on cancer-related internet search activity has not been well studied. Breast (BC), Lung (LC) and Colorectal Cancers (CRC) are the leading causes of cancer incidence and mortality among women [4] and have their respective CAMs during October, November and March [5].

Using Google Trends, a public web facility of Google Inc. based on Google Search, we compared the relative frequency of search of terms ‘Breast Cancer’,‘Lung Cancer’ and ‘Colon Cancer’ between 1st January 2004 and 31st January 2017 (n=158 months). The program assigns a reference value of 100 for the point of maximum popularity from among the search terms, and provides relative monthly scores for all terms, which we termed interest scores (IS). IS were then compared among cancers for the overall period (n=158 months) and specifically during their CAMs (n=13 months). Within each cancer, IS were then compared during the CAMs (n=13 months) as compared to the remaining months (n=145 months). Parametric and non-parametric analyses were carried out (wherever applicable) using ANOVA and Kruskal-Wallis tests respectively. A *P*-value of <.05 was considered significant.

We found that BC had higher IS (mean± S.D) than LC and CRC for the entire study period (38.83±14.46 vs14.71 ±4.56 and 11.98±2.13 respectively, *P*<0.001*), including a peak IS of 100 in October, 2004. BC also had significantly higher IS during its CAM (October) than the CAMs for LC (November) and CRC (March); 69.92±11.75 vs 15.38±4.54 and 13.53±2.43 respectively, *P*<0.001*. While BC (69.92±11.02 vs 36.04±11.02; *P*<0.001*) and CRC (13.53±11.84 vs 11.85 ± 2.06; *P*=0.036*) had higher IS during their CAMs as compared to other months, LC did not (15.38 ±4.53 vs 14.65±4.57; *P*=0.3019) (Table 1).

We concluded that ongoing campaigns for BC awareness are very effective at driving internet search activity, not only at baseline (2.5-3 times) but even more so also during its CAM (4-5 times) as compared to the other two leading malignancies among women (CRC and LC). Despite having a higher mortality than CRC, the campaign for LC was unable to significantly impact internet search activity during its CAM. Reasons behind the success of the BC awareness campaign in driving internet search activity should be further explored and applied to those for other malignancies such as LC and CRC, which also continue to have high mortality. Therefore, as highlighted by Ling et al [1], the use of infoveillance can serve as a cost-effective solution to evaluate the effects of health campaigns. However, further research is needed to definitively establish Google Trends as a valid and reliable tool for this purpose.
